# Half-Dose versus Single-Dose Gadobutrol for Extracellular Volume Measurements in Cardiac Magnetic Resonance

**DOI:** 10.3390/jcdd10080316

**Published:** 2023-07-26

**Authors:** Patrick Doeblin, Fridolin Steinbeis, Martin Witzenrath, Djawid Hashemi, Wensu Chen, Karl Jakob Weiss, Philipp Stawowy, Sebastian Kelle

**Affiliations:** 1 Department of Cardiology, Angiology and Intensive Care Medicine, Deutsches Herzzentrum der Charité, Augustenburger Platz 1, 13353 Berlin, Germany; 2 German Center for Cardiovascular Research (DZHK), Partner Site Berlin, 10785 Berlin, Germany; 3 Charité—Universitätsmedizin Berlin, Corporate Member of Freie Universität Berlin and Humboldt-Universität zu Berlin, Charitéplatz 1, 10117 Berlin, Germany; 4 Department of Infectious Diseases and Respiratory Medicine, Charité—Universitätsmedizin Berlin, Corporate Member of Freie Universität Berlin, Humboldt-Universität zu Berlin, 10117 Berlin, Germany; 5 German Center for Lung Research (DZL), 10117 Berlin, Germany; 6 Berlin Institute of Health at Charité—Universitätsmedizin Berlin, Charitéplatz 1, 10117 Berlin, Germany

**Keywords:** ECV, CMR, gadobutrol, renal insufficiency, T1 mapping, cardiac, gadolinium

## Abstract

Background: Cardiac magnetic resonance (CMR) imaging with gadolinium-based contrast agents offers unique non-invasive insights into cardiac tissue composition. Myocardial extracellular volume (ECV) has evolved as an objective and robust parameter with broad diagnostic and prognostic implications. For the gadolinium compound gadobutrol, the recommended dose for cardiac imaging, including ECV measurements, is 0.1 mmol/kg (single dose). This dose was optimized for late enhancement imaging, a measure of focal fibrosis. Whether a lower dose is sufficient for ECV measurements is unknown. We aim to evaluate the accuracy of ECV measurements using a half dose of 0.05 mmol/kg gadobutrol compared to the standard single dose of 0.1 mmol/kg. Methods and results: From a contemporary trial (NCT04747366, registered 10 February 2021), a total of 25 examinations with available T1 mapping before and after 0.05 and 0.1 mmol/kg gadobutrol were analyzed. ECV values were calculated automatically from pre- and post-contrast T1 relaxation times. T1 and ECV Measurements were performed in the midventricular septum. ECV values after 0.05 and 0.1 mmol/kg gadobutrol were correlated (R^2^ = 0.920, *p* < 0.001). ECV values after 0.05 mmol/kg had a bias of +0.9% (95%-CI [0.4; 1.4], *p* = 0.002) compared to 0.1 mmol/kg gadobutrol, with limits of agreement from −1.5 to 3.3%. Conclusions: CMR with a half dose of 0.05 mmol/kg gadobutrol overestimated ECV by 0.9% compared with a full dose of 0.1 mmol/kg, necessitating adjustment of normal values when using half-dose ECV imaging.

## 1. Introduction

Cardiac magnetic resonance (CMR) imaging offers unique non-invasive insights into cardiac tissue composition. Expansion of the tissue extracellular volume (ECV) can be quantified using pre- and post-contrast T1 relaxation times of blood and myocardium [1,2]. Myocardial ECV has evolved as an objective and robust parameter with broad diagnostic and prognostic implications [3,4,5,6,7,8]. While gadolinium-based contrast agents generally have a favorable safety profile, and the risk of nephrogenic systemic fibrosis has been mostly eliminated by the use of group II gadolinium-based contrast agents, it is recommends to use the smallest dose possible to provide the necessary information [9,10,11]. For the gadolinium compound gadobutrol, the recommended gadolinium dose for cardiac imaging, including ECV measurements, is 0.1 mmol/kg [12,13]. This dose was optimized for late enhancement imaging, a measure of focal fibrosis. Whether a lower dose is sufficient for ECV measurements is unknown. The objective of this study is to compare ECV measurements using a gadolinium dose of 0.05 mmol/kg to a dose of 0.1 mmol/kg.

## 2. Materials and Methods

From a contemporary trial (NAPKON-HAP, NCT04747366, registered 10 February 2021) [14], a secondary analysis of all CMR examinations performed at our center was performed with the following inclusion criteria: Two bolus applications of contrast agent (for stress and rest perfusion), use of 0.05 mmol/kg gadobutrol (Gadovist^®^, Bayer Healthcare, Leverkusen, Germany) for each bolus, and acquisition of T1 maps before each bolus, as well as at the time of late enhancement imaging. 25 examinations were identified. Details on the inclusion criteria are given in the Supplementary Materials.

**Figure 1:**
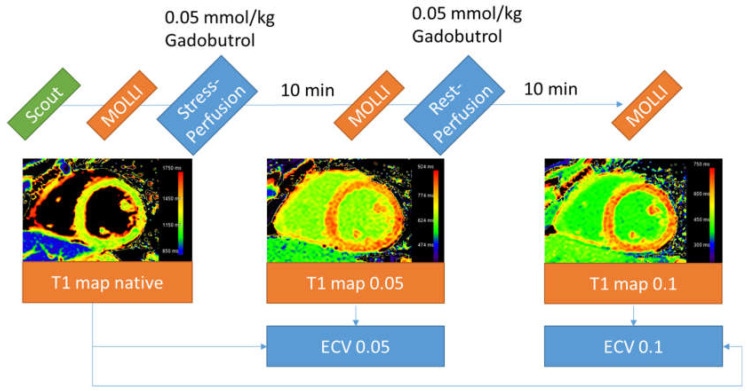
Timeline of the CMR protocol. Sequences not relevant to this analysis were omitted for clarity. The color scale of the T1 map after the first perfusion was manually adjusted. ECV—extracellular volume; MOLLI—modified Look–Locker inversion.

The secondary analysis was approved by the ethics committee of the Charité–Universitätsmedizin Berlin. All patients had previously suffered from COVID-19 and underwent research CMR to assess cardiac complications. The study protocol included quantitative analysis of myocardial stress and rest perfusion 10 min apart, requiring T1 mapping before each contrast bolus. Additionally, T1 mapping was performed 10 minutes after the second bolus for standard ECV calculations. A schematic timeline of the protocol is given in Figure 1. The full CMR protocol is shown in the Supplementary Materials. 

### 2.1. Image Acquisition

All patients were examined with a clinical 3 Tesla MRI scanner (Ingenia, Philips Healthcare, Best, The Netherlands) equipped with a body receiver coil. T1 mapping was performed using a modified Look–Locker (MOLLI) 5s(3s)3s—scheme [15]. Typical imaging parameters were as follows: acquired voxel size = 1.97 × 2.00 × 10 mm^3^, reconstructed voxel size = 1.17 × 1.17 × 10 mm^3^, balanced SSFP readout, flip angle = 20°.

### 2.2. Image Analysis

Image analysis was performed offline using commercially available software (IntelliSpace Portal, Version 12.1, Philips Medical Systems Nederland B.V., Best, The Netherlands). MOLLI images were automatically corrected for in-plane motion. T1 relaxation time measurements were performed in the midventricular septum and ECV values calculated from pre- and post-contrast T1 relaxation times. T1 measurements were performed independently by two experienced readers (PD and JW, both level 3 CMR-certified).

### 2.3. Statistical Analysis

Statistical analysis was performed using IBM^®^ SPSS 25^®^ (IBM, Armonk, NY, USA) Baseline data was reported as mean ± standard deviation (SD) for interval and ratio-scaled parameters and as number and percentage for nominal and ordinal-scaled parameters. ECV measurements were compared using a Pearson correlation, a Bland–Altman analysis, and a paired *t*-test. 

## 3. Results

**Figure 2:**
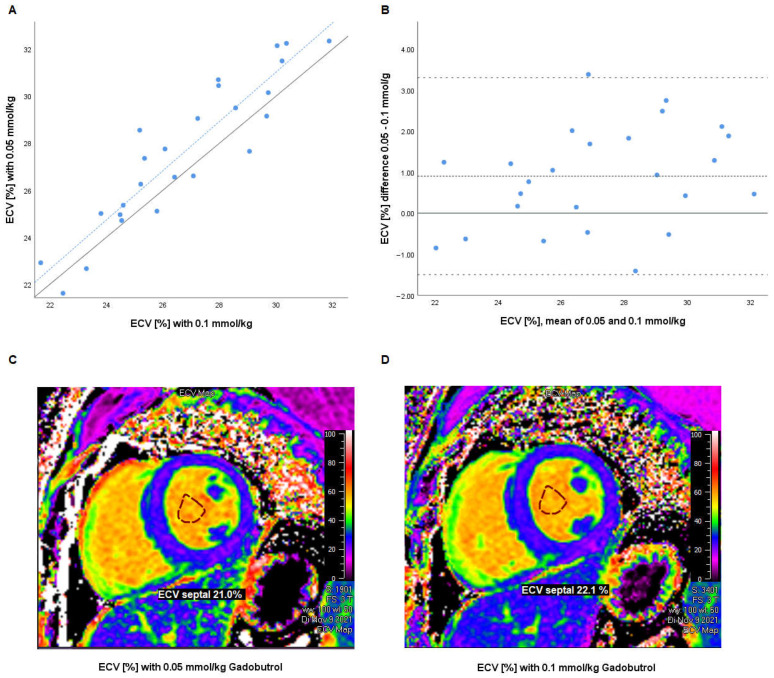
(**A**) Scatter plot of ECV measurements with 0.05 and 0.1 mmol/kg gadobutrol. Grey line: line of identity (y = x). Dotted blue line: regression line (R^2^ = 0.846, *p* < 0.001). (**B**) Bland–Altman analysis of intraindividual differences (0.1–0.05 mmol/kg) in percentage points versus mean of both measurements. Dashed line: mean difference (bias). Pointed line: limits of agreement (mean ± 1.96 SD of difference). (**C**,**D**) Representative ECV maps with (**C**) 0.05 mmol/kg and (**D**) 0.1 mmol/kg gadobutrol (created using Medis Suite MRCT 2021, Medis Medical Imaging Systems bv, Leiden, the Netherlands) for demonstrative purposes only; measurements were performed using Philips IntelliSpace Portal). ECV—extracellular volume, SD—standard deviation.

Twenty-five patients with available T1 mapping sequences at all three time points were included. Basic demographic parameters and CMR results are given in Table 1. A Bland–Altman plot of ECV values 10 min after the first dose of 0.05 mmol/kg and 10 min after the second dose of 0.05 mmol/kg (for a total dose of 0.1 mmol/kg) is depicted in Figure 2. ECV values after 0.05 and 0.1 mmol/kg gadobutrol were correlated (r = 0.920, *p* < 0.001). ECV values after 0.05 mmol/kg gadobutrol had a bias of +1.1% (95%-CI [0.4; 1.4], *p* = 0.002) compared to 0.1 mmol/kg gadobutrol, with limits of agreement from −1.5 to 3.3%. There was no correlation between bias and hematocrit (r = −0.220, *p* = 0.291).

## 4. Discussion

We compared a half dose of 0.05 mmol/kg gadobutrol with the standard single dose of 0.1 mmol/kg for myocardial ECV measurements. The bias was +0.9 ± 1.2% (95% CI: [0.4; 1.4], *p* = 0.002) with limits of agreement from −1.5 to 3.3%.

These intraindividual differences are in the range of values reported in studies comparing different scanners and different protocols [16,17]. Previous studies found significantly higher ECV values after 0.1 compared to 0.2 mmol/kg gadobutrol [18,19].

Studies comparing the ECV at different time intervals after gadolinium application found constant to slightly increasing values at later time points [16,19,20]. Miller et al. found increases in ECV of 1.5%, 1.2%, and 1.0% from 2 to 20 min post-contrast for 0.1, 0.15, and 0.2 mmol/kg [19]. This is in line with our findings, as longer intervals translate to lower contrast agent concentrations due to renal elimination. Our study adds to the existing evidence that the presumptions underlying the ECV calculation might be incomplete, as the contrast dose should not influence the calculated ECV. One possible explanation would be the known non-linearity in the measurement errors of the MOLLI T1-mapping sequence [15]. Another theoretical possibility, albeit without current experimental evidence, could be a non-linearity in the contrast agent relaxivity in blood pool or myocardium.

While a dose of at least 0.1 mmol/kg should be considered standard for ECV imaging, including in patients with severe kidney disease, our data suggest that a reduced dose of 0.05 mmol/kg may still offer sufficient diagnostic accuracy to exclude large alterations in ECV.

Limitations

Due to the small sample size, our results should be considered hypothesis-generating and need to be validated in a larger cohort of with different pathologies. Our study compared a single bolus of 0.05 mmol/kg to a split bolus of two times 0.05 mmol/kg gadolinium as reference standard. McDiarmid et al. have addressed the comparability of ECV measurements between split- and single-bolus examinations and found no systematic difference [17]. Another possible confounder in our study is the use of vasodilator stress during the first contrast bolus, as heart rate and myocardial vasodilation are known to influence myocardial relaxation times. As the heart rates were very similar at the time of T1 mapping after the first and second bolus (Table 1), any differences in vasodilator effect are probably negligible. 

## Figures and Tables

**Table 1:** Basic demographic parameters and CMR results.

	Total (N = 25)
Age	59.4 ± 15
Sex (male)	12 (52%)
Hematocrit [%]	42.0 ± 4.7
LV-EDVi [mL/m²]	73.0 ± 13.3
LV-EF [%]	60.0 ± 6.8
RV-EDVi [mL/m²]	73.6 ± 14.0
RV-EF [%]	56.3 ± 8.4
Native	
HR [s^−1^]	67.8 ± 9.2
T1 myo [ms]	1256 ± 39
T1 blood [ms]	1859 ± 85
Half dose (0.05 mmol/kg)	
HR [s^−1^]	73.6 ± 8.3
dT [mm:ss]	10:15 ± 02:06
T1 myo [ms]	786.6 ± 51.7
T1 blood [ms]	654 ± 74
ECV [%]	27.6 ± 3.1
Full dose (0.1 mmol/kg)	
HR [s^−1^]	72.7 ± 7.6
dT [mm:ss]	10:54 ± 2:10
T1 myo [ms]	608 ± 39
T1 blood [ms]	422 ± 48
ECV [%]	26.7 ± 2.7

Myocardial (myo) and blood pool (blood) T1 relaxation times before contrast application (native), after the first perfusion (half dose, 0.05 mmol/kg), and after the second perfusion (full dose, total of 0.1 mmol/kg). dT time between the perfusion scan and the T1 mapping scan, ECV extracellular volume, HR heart rate. Continuous values as mean ± standard deviation, nominal values as number and percentage. T1 mapping and ECV mean of both readers.

## Data Availability

The data are available upon reasonable request.

## Supplementary Materials

The following supporting information can be downloaded at: https://www.mdpi.com/article/10.3390/jcdd10080316/s1. Patient selection; Full CMR-Protocoll of the NAPKON-HAP-Trial, local Study Site (DHZB); Inter-Observer-Variablity.
