# Supplementary material for: Half-Dose versus Single-Dose Gadobutrol for Extracellular Volume Measurements in Cardiac Magnetic Resonance
Source: J Cardiovasc Dev Dis. 2023 Jul 26;10(8):316. doi: 10.3390/jcdd10080316 (PMC10455162; doi:10.3390/jcdd10080316)
Supplement: Supplementary file 1 [file jcdd-10-00316-s001.zip › jcdd-2430217-supplementary.pdf]

# Half Dose versus Single Dose Gadobutrol for Extracellular Volume Measurements in Cardiac Magnetic Resonance

Supplementaray Material

Patient selection ....................................................................................................................2
Full CMR-Protocoll of the NAPKON-HAP-Trial, local Study Site (DHZB)...........................................3
Inter-Observer-Variability ...........................................................................................................4

## Patient selection

All examinations performed in our hospital between May 2021 and December 2022 as part of the NAPKON-HAP-trial were screened for the following inclusion criteria:

1. Aqcuisition of stress and rest perfusion with a bolus of 0.05 mmol/kg gadobutrol each
2. Aqcuisition of T1-mapping native, after the first bolus and after the second bolus in diagnostic quality.
3. Availability of an hematocrit at the day of the CMR
4. If both baseline and follow-up-examinations are available for a patient, only the baseline examination was included.

41 CMR examinations were identified in the specified time frame.

12 Examinations were excluded as either no contrast agent was given, only one bolus was given, or a different contrast agent dose was given.

2 Examination were excluded due to incomplete or non-diagnostic T1-mapping sequences.

1 Examination was excluded due to missing hematocrit information.

1 Examination was excluded as it was a follow-up examination of a patient already included.

25 Examinations were available for analysis.

Full CMR-Protocoll of the NAPKON-HAP-Trial, local Study Site (DHZB)

| Time (min) | Description | Sequence Name | Orientation |
|---|---|---|---|
| -18 | Survey | FFE | Transversal, Sagital, Coronal |
| -15 | Planning | Cine | RAO, p4CV |
| -13 | LV 3CV | Cine, 50 Phases per Beat | 3CV |
| -12 | Native T1-Mapping | MOLLI (3-3-5) | SAX 2 slices (basal, medial)<br>Slice thickness 10mm |
| -8 | T2-weighted imaging | T2 STIR Black Blood TR 2000ms, TE 75ms | SAX full coverage (slice thickness 8mm, no gap), 4CV, 2CV, 3CV |
| -4 | T2-Mapping | GraSE | SAX 2 slices (basal, medial)<br>Slice thickness 8mm |
| 0 | Quantitative Vasodilator Stress-Perfusion | Qperf | SAX 3 slices (basal, medial, apical)<br>Slice 4 in ascending Aorta |
| 3 | LV SAX, 4CV, 2CV | Cine, 50 Phases per Beat | SAX full coverage (slice thickness 8mm, no gap), 4CV, 2CV |
| 8 | Real-Time in-/expiration | sBFFE | Medial SAX |
|  | T1-Mapping | MOLLI (3-3-5) | SAX 1 slice |
| 10 | Quantitative Rest-Perfusion | Qperf | SAX 3 slices (basal, medial, apical)<br>Slice 4 in ascending Aorta |
| 12 | RV-Function | Cine, 25 Phases per Beat | Transversal full stack (slice thickness 8mm, no gap) |
| 17 | 2D-Flow | Phase Contrast VENC 200 cm/s (higher if aliasing) | Ascending aorta & pulmonary artery |
| 20 | Late gadolinium enhancement (LGE) | mDIXON | SAX full coverage (slice thickness 10mm, gap -5mm), 4CV, 2CV, 3CV |
| 25 | Post-contrast T1-Mapping | MOLLI (3-3-5) | SAX 2 slices (basal, medial)<br>Slice thickness 10mm |
| 27 | Myocardial strain | fSENC | SAX 3 slices (basal, medial, apical), 4CV, 2CV, 3CV |
| 32 | End (total 50 min) |  |  |

Inter-Observer-Variability

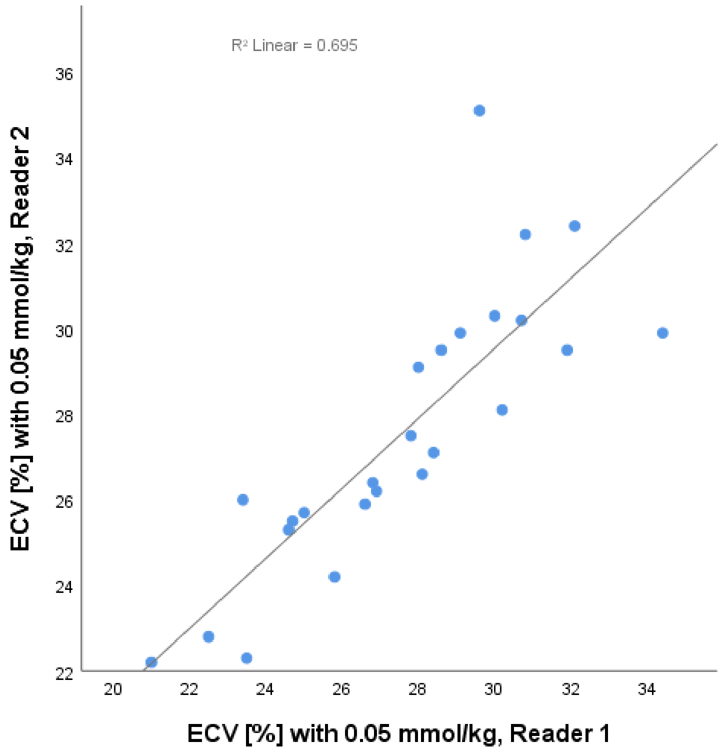


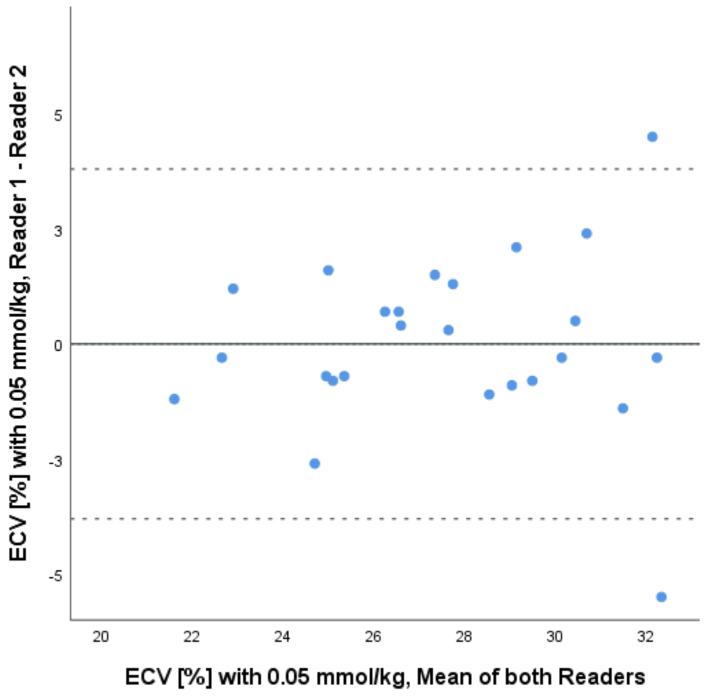

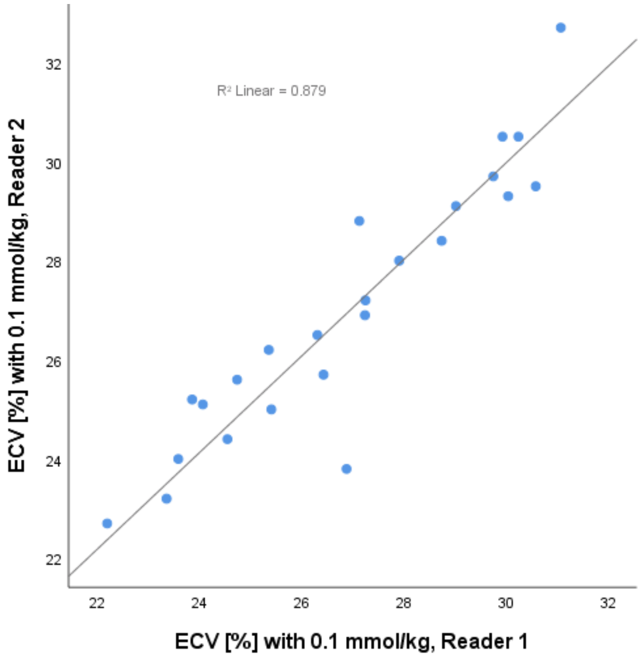
R² Linear = 0.879
ECV [%] with 0.1 mmol/kg, Reader 2
ECV [%] with 0.1 mmol/kg, Reader 1
22
24
26
28
30
32

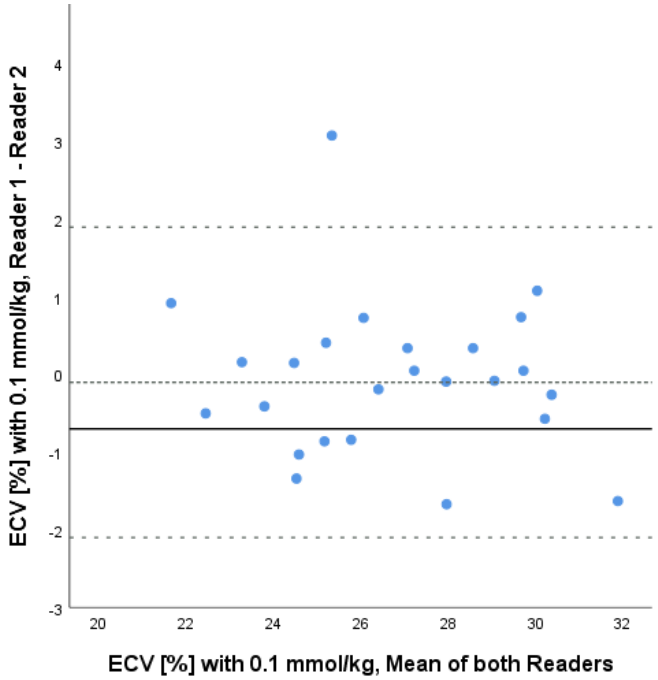
ECV [%] with 0.1 mmol/kg, Reader 1 - Reader 2
ECV [%] with 0.1 mmol/kg, Mean of both Readers
4
3
2
1
0
-1
-2
-3
20
22
24
26
28
30
32
